# Post-concussion syndrome among patients experiencing head injury attending emergency department of Hawassa University Comprehensive specialized hospital, Hawassa, southern Ethiopia

**DOI:** 10.1186/s10194-018-0945-0

**Published:** 2018-11-21

**Authors:** Asres Bedaso, Ephrem Geja, Mohammed Ayalew, Zewdie Oltaye, Bereket Duko

**Affiliations:** College of Medicine and Health Sciences, School of Nursing, Hawassa University, P.O.BOX: 1560 Hawassa, Ethiopia

**Keywords:** Post-concussion syndrome, Mild traumatic brain injury, Head injury, Hawassa

## Abstract

**Introduction:**

Post-concussion syndrome (PCS) is defined as the presence of 3 or more of the following signs and symptoms after experiencing head injury such as headache, dizziness, fatigue, irritability, insomnia, difficulty of concentration or memory difficulty. In Ethiopia, even though there was no research conducted on post-concussion syndrome, it is common health problems after experiencing head trauma that affect the productive age group, which directly or indirectly influences the development of the country.

**Objective:**

To assess the prevalence and determinants of post-concussion syndrome among patients experiencing head injury attending emergency department of Hawassa University Comprehensive Specialized hospital, Hawassa, Southern Ethiopia.

**Methods:**

Institution based cross sectional study was conducted from November 1, 2017 to March 30, 2018, in Hawassa University Comprehensive Specialized Hospital emergency department, Hawassa, Southern Ethiopia.

**Result:**

A total of 275 cases were interviewed during data collection period with response rate of 95.2%. More than half (55.7%) of patients were within age range of 25–34 and Majorities (55.6%) of patients were married. About two-fifths of study participants (41.5%) had at least three symptoms of post-concussion syndrome components. Headache and restlessness were the most symptoms occurring in varying severity while double vision and fatigue were less severe among others. Occupation, cause of injury and location of injury were significant determinants of post-concussion syndrome.

**Conclusion:**

About 41.5% of study participants had at least three symptoms of PCS. Occupation, cause of injury and location of injury were significantly associated with the occurrence of PCS.

**Electronic supplementary material:**

The online version of this article (10.1186/s10194-018-0945-0) contains supplementary material, which is available to authorized users.

## Introduction

According to World Health Organization (WHO) Post-concussion syndrome (PCS) is defined as the presence of 3 or more of the following signs and symptoms after experiencing head injury such as headache, dizziness, fatigue, irritability, insomnia, difficulty of concentration, or memory difficulty [1].The diagnosis of PCS is made in people who have suffered with a head trauma when persistent post-traumatic amnesia, loss of consciousness, or post-traumatic seizures and neuropsychological impairment must be present for three months after the injury which must have been absent or less severe before the injury [2].

PCS is one of the commonest neuropsychiatric consequence after having traumatic brain injury (TBI) [3] and its onset is more often for the period of the first month after trauma, but it may continue for months even years or more after head trauma [1, 3]. The magnitude of PCS differs, however most studies reported that about 15% of individuals with a history of a single concussion develop persistent symptoms associated with the injury and these symptoms may continue last for more than three months after the head injury [4]. However, there is substantial risk of misdiagnosis of PCS, because most of the symptoms related to PCS are common or may be worsen by other disorders. For example; headaches that occur after a concussion may feel like migraine or tension-type headaches [5].

Some of the risk factors that have been associated with PCS are pre-existing medical or psychological conditions, expectations of disability, being female and older age. Physiological and psychological factors present before, during, and after the injury are all thought to be involved in the development of PCS [6] and the questions of the cause or causes of PCS have been a lot debated for many years and remain controversial [7]. Some experts found out that post-concussion symptoms are caused by structural damage to the brain or disruption of neurotransmitter systems [8]. Though, about 38% of people who experience a head injury with symptoms of concussion had no radiological evidence of brain lesions [9].

In addition, about 10% of people with PCS develop sensitivity to light or noise, about 5% experience a decreased sense of taste or smell, and about 14% report blurred vision [10] and some people may also have double vision or ringing in the ears [11].

Post-concussion syndrome (PCS), the most common entity to be diagnosed in people who have experienced TBI, is a constellation of physical, cognitive, emotional, and behavioral symptoms and its prevalence varies from 11% to 64% [12]. The prevalence of persistent symptoms at 1 year is not known, but is estimated to be 5%. There is limited information on the prevalence and incidence of PCS in children. Despite the prevalence of PCS, uncertainty exists about the validity of this diagnosis because behavioral disturbances frequently occur in children after any injury and because factors present before injury and medico legal concerns after injury may influence recovery [13]. Up to 60% of patients with minor traumatic brain injury (MTBI) will suffer from post-concussion syndrome (PCS) characterized by persistence of symptoms such as headache, fatigue, memory difficulties and emotional liability [14].

According to different researches which were done worldwide, head injuries are common health problems of the world which affects mainly productive age groups of the world and contributes strongly to costs in the health care system. In Africa, head injuries are the common health problems and are substantial causes for morbidity and mortality which mostly affects productive age group of the population and brings economic consequences for both individuals and society of the continent [15].

In Ethiopia, even though there was no research conducted on post-concussion syndrome, it is a common health problem that affect the productive age group, which directly or indirectly influences the development of the country [16]. So, since the problem affects the productive age group of the country, paying attention to carry out this study assessing the magnitude and determinants of post-concussion syndrome is most crucial.

## Methods

### Study design, study area and study period

Institutional based cross-sectional study was conducted in Emergency department of Hawassa University Comprehensive and Specialized Hospital (HUCSH), from November 1, 2017 to March 30, 2018 which is found in Hawassa city 273 Km far south from Addis Ababa. HUCSH started giving service since 2005 and serves for more than 18 million people of the catchment area, from the southern region and the surrounding Oromia zones. The hospital had 400 beds for inpatient service and also gives different outpatient services.

### Population

All patients presented to adult emergency department of HUCSH with head trauma were the source population and all head injury patients presented to adult emergency department in HUCSH during the study period were the study population.

### Inclusion and exclusion criteria

All patients experiencing head injury whose age 18 year or above were included in the study and patients who were severely ill or unconscious were excluded from the study.

### Sample size and sampling technique

The sample size was determined using single population proportion formula considering the following assumptions: confidence interval (CI) of 95% (*Z* = 1.96), absolute precision or tolerable margin of error (*d* = 0.05), and anticipated proportion of patients who experience PCS =50% (*P*). Since the source population was < 10,000, we used a correction formula, adding 10% non-response rate the final sample size was 289. Consecutive sampling technique was used to select the respondents for interview.

### Data collection instrument

In order to assess symptoms after experiencing head injury, a number of measures have been described. One commonly used tool is the Rivermead Post-Concussion Symptoms Questionnaire (RPQ), which has demonstrated reliability and validity [17, 18]. This questionnaire takes into account the high background prevalence of symptoms by asking the patients not only if symptoms are present, but also to rate the intensity of each of 16 symptoms as compared to before the Mild Traumatic Brain injury (MTBI).

### Data quality control measures

The data was collected by trained nurse professionals and supervised by two emergency and critical care nurse specialists. The questionnaire was pre tested on 5% of the study participant prior to the actual data collection period for its clarity, understandability and completeness. The data collected during the pre-test was not included in the final analysis.

### Data processing and analysis

The collected data were checked cleaned, coded and entered and analyzed using SPSS version 21. Descriptive statistical analysis was used to estimate the frequencies and percentages of the variables. Bivariate and multivariate logistic regression analysis was used to see the association between outcome and explanatory variables. The strength of the association was measured by odds ratio with 95% CI and *P*-value less than 0.05 was considered as statistically significant.

## Results

A total of 275 cases were interviewed over five months data collection period with final response rate of 95.2%. More than half (55.7%) of patients were in age range of 25–34. Majorities (55.6%) of patients were married and 74.9% were living with family (Table 1).

**Table 1:** Socio-demographic characteristics of study participants attending emergency outpatient department of HUCSH, Hawassa, Southern Ethiopia (*n* = 289)

Characteristics	Categories	Number	Percent
Age	18–24	61	22.2
	25–29	100	36.4
	30–34	53	19.3
	35–39	38	13.8
	40+	23	8.4
Sex	Male	161	58.5
	Female	114	41.5
Religion	Orthodox	99	36.0
	Muslim	64	23.3
	Protestant	89	32.4
	Catholic	22	8.4
	Other	1	0.4
Marital status	Single	105	38.2
	Married	153	55.6
	Divorced	6	2.2
	Widowed	11	4.0
Occupation	Government employee	84	30.5
	Merchant	72	26.2
	Farmer	34	12.4
	Student	44	16.
	Daily labor	15	5.5
	House wife	26	9.5
Educational status	Unable to read and write	43	15.6
	Able to read and write	39	14.2
	Primary	31	11.3
	Secondary	141	51.3
	College and above	21	7.6
Ethnicity	Sidama	104	37.8
	Wolaita	35	12.7
	Amhara	39	14.2
	Oromo	62	22.5
	Tigre	2	0.7
	Gurage	19	6.9
	Others	14	5.1
Living condition	With family	206	74.9
	Alone	69	25.1

### Social support and causes of injury

From the total study participants 31.6% of respondents have very good social support **(**Additional file 1).43.6% of study participants get injured due to motor bicycle accident (Additional file 2).

### Characteristics of injury and symptoms of PCS

From the study participants 65.1% had < 1 week duration of illness and 30.9% faced injury on the right lateral part of their brain (Table 2). Among the study participants 227(82.5%) complains headache as a major symptom of PCS (Additional file 3).

**Table 2:** Characteristics of injures of study participants attending emergency outpatient department of Hawassa University compressive specialized hospital, Hawassa, Southern Ethiopia (*n* = 289)

Characteristics	Categories	Number	Percent
Duration of illness	< 1 week	179	65.1
	1–2 weeks	55	20.0
	34 weeks	32	11.6
	> 1 month	9	3.3
Location of head injury	Right lateral	85	30.9
	Left lateral	86	31.3
	Frontal	75	27.3
	Other site	29	10.5
Clinical presentation at time of admission	Shock	55	20.0
	Unconscious	115	41.8
	Bleeding	77	28.0
	Other	28	10.2
Length of stay in hospital	< 5 days	108	39.3
	1 week	87	31.6
	2–4 weeks	61	22.2
	5+ weeks	19	6.9
Duration of treatment	< 1 week	123	44.7
	1–5 weeks	115	41.8
	6–10 weeks	26	9.5
	10+ weeks	11	4.0

### Prevalence of post-concussion syndrome

We computed total score from 16 items, each measured from zero to four. All values were summed, with expected value from 0 to 64. The finding showed that mean of 7.57 with SD + 2.63. In this study the overall prevalence of post-concussion syndrome is 41.5%.

### Factors associated with PCS

During Multivariate logistic regression being student (*P* = 0.04, CI [0.14, 0.95]), Motor bicycle as a cause of injury (*P* = 0.04, CI [0.30, 0.98]) and location of head injury (*P* = 0.02, CI [0.09, 0.83]) were significantly associated with post-concussion syndrome (Table 3).

**Table 3:** Factors Associated PCS (Bivariate and Multivariate logistic regression) of study participants attending emergency outpatient department of Hawassa university compressive specialized hospital, Hawassa, Southern Ethiopia (*n* = 289)

Variable	PCS		COR (95% CI)	AOR (95% CI)	*P*-Value
	**No**	Yes			
Age					
18–24	38	23	1	1	
25–29	64	36	0.9 (0.48–1.79)	0.61 (0.28–1.35)	0.22
30–34	32	21	1.08 (0.51–2.31)	0.66 (0.26–1.70)	0.39
35–39	19	19	1.65 (0.73–3.75)	1.02 (0.36–2.88)	0.97
> 40	8	15	3.09 (1.14–8.44)	2.01 (0.57–7.09)	0.28
Sex					
M	86	75	1		
F	75	39	0.59(0.36–0.98)	0.62 (0.34–1.12)	0.11
Marital status					
Single	67	38	1	1	1
Married	87	66	1.34(0.80–2.23)	1.03 (0.52–2.03)	0.93
Divorced\widowed	7	10	2.53 (0.88–7.16)	1.94 (0.55–6.77)	0.30
Occupation					
Government employee	45	39	1	1	1
Merchant	41	31	0.87(0.46–1.6)	0.62 (0.29–1.30)	0.21
Farmer	18	16	1.02 (0.46–2.2)	0.41 (0.13–1.26)	0.12
Student	31	13	0.48(0.22–1.05)	0.36 (0.14–0.95)*	0.04*
Day labor	10	5	0.57 (0.18–1.83)	0.25 (0.06–1.06)	0.06
House wife	16	10	0.72 (0.29–1.77)	0.36 (0.10–1.28)	0.11
Education					
Unable to write & read	20	23	1	1	1
Read & write only	23	16	0.61 (025–1.4)	0.61 (0.25–1.45)	0.26
Primary	19	12	0.55 (0.22–1.4)	0.55 (0.22–1.40)	0.21
Secondary	88	53	0.52 (0.26–1.04)	0.52 (0.26–1.04)	0.66
College & above	11	10	0.79 (0.27–2.25)	0.79 (0.28–2.25)	0.66
Social support					
Excellent	54	32	1	1	
Very good	50	37	1.24(0.67–2.29)	1.28 (0.65–2.50)	0.48
Good	41	27	1.11 (0.57–2.1)	1.13 (0.55–2.31)	0.74
Fair & poor	16	18	2.03 (0.89–45)	1.58 (0.63–4.01)	0.33
Cause of head injury					
Car	56	52	1	1	1
Motor bicycle	77	43	0.6 (0.35–1.02)	0.54 (0.30–0.98)	0.04*
Fall accident	23	14	0.66 (0.31–1.4)	0.94 (0.39–2.27)	0.90
Other	5	5	1.07 (029–0.9)	1.98 (0.47–8.37)	0.35
Location of head injury					
Rt Lateral	48	37	1	1	1
Lt Lateral	48	38	1.03 (0.561–1.8)	1.01 (0.52–1.96)	0.97
Frontal	42	33	1.02 (0.55–1.9)	1.02 (0.51–2.05)	0.95
Other site**	23	6	0.34 (0.12–0.9)	0.28 (0.09–0.83)	0.02*

* P value *<* 0.05 (Significant Association), ** Back, occipital and parietal area

## Discussion

This study was designed to assess prevalence of PCS and its associated factors among patients experiencing head injury and attending emergency department in Hawassa university comprehensive specialized hospital in 2018.

The Overall prevalence of PCS among patients experiencing head injury and attending emergency department in Hawassa university comprehensive specialized hospital was 41.5%. The finding of this study was higher than study done in New Zealand where more than 30% of participants reported PCS symptoms [19]. In addition it was significantly higher as compared to Malaysian [20] and Swedish [21] study which was 8.1% and 34%of patients survived from mild TBI developed PCS, respectively. The variation between the current study and the study mentioned above might be due to the difference in sample size, studies area and socioeconomic difference among study participants.

Students were 36% less likely to develop PCS compared to government employee. As it was evidenced, among study participants who experience head injury 30.5% and 16% were government employee and students by occupation respectively. This implies those who experienced head injury are more likely to develop PCS. Also, as it was commonly observed in the study area government employees uses motor bicycle compared with students, which will increase risk of road traffic accident.

Patients injured by motor bicycle were 54% less likely to develop PCS compared with those injured due to car accident. This might be due to the fact that it is obligatory to use helmets by motor bicycle drivers which protect them from head injury.

Patients who injured occipital area of the brain were 28% less likely to develop PCS compared to with those who injured on right lateral. This finding is also supported by the follow up study conducted in USA, study participants who injured on fronto temporal and parietal area were severely affected and atrophied because of head injury compared with injuries on the different part of the brain [22].

## Conclusion

About 41.5% of study participants had at least three symptoms of PCS. Occupation, cause of injury and location of injury were significantly associated with the occurrence of PCS.

## Recommendation

Future studies better to consider imaging finding of individuals with head injury as supportive data in addition to assessing symptoms included in the PCS tool.

### Limitation

Since the study design is cross-sectional, it does not allow inferring causation.

## Additional files

Additional file 1: Social support of study participants attending emergency outpatient department of Hawassa University comprehensive specialized hospital Hawassa, Southern Ethiopia (*n* = 289) (DOCX 25 kb)

Additional file 2: Cause of injures are the study participant attending emergency outpatient department of Hawassa university comprehensive specialized hospital Hawassa, Southern Ethiopia (*n* = 289) (DOCX 24 kb)

Additional file 3: Symptoms of PCS of study participants attending emergency outpatient department of Hawassa University comprehensive specialized hospital, Hawassa, Southern Ethiopia (*n* = 289) (DOCX 16 kb)

## Abbreviations

ACRM: American congruent of rehabilitation medicine
CDC: Center for disease control
CI: Confidence Interval
DHS: Demographic and Health Survey
DSM: Diagnostic and statistical manual
ED: Emergency department
GCS: Glasgow coma scale
HUCSH: Hawassa University compressive specialized hospital
ICD: International classification of diseases
ICU: Intensive care unite
MTBI: Mild traumatic brain injuries
NGO’s: Non-Governmental Organization
NICU: Neonatal intensive care unit
PCD: Post-concessional disorder
PCS: Post-concussion syndrome
PCSS: Post-concussion syndrome scale
PTA: Post traumatic amnesia
RTA: Rode traffic accident
SD: Standard deviation
TBI: Traumatic brain injuries
US: United state

## References

[1] Mittenberg WSS (2000). Diagnosis of mild head injury and the postconcussion syndrome. J Head Trauma Rehabil.

[2] Association AP (1994). diagnostic and statistical manual of mental disorders.

[3] Jorge Ricardo E (2005). Neuropsychiatric consequences of traumatic brain injury: a review of recent findings. Current Opinion in Psychiatry.

[4] McHugh T, Laforce R, Gallagher P, Quinn S, Diggle PBL (2006). Natural history of the long-term cognitive, affective, and physical sequelae of a minor traumatic brain injury. Brain Cogn.

[5] Bigler ED (2008). Neuropsychology and clinical neuroscience of persistent post-concussive syndrome. Int Neuropsychol Soc.

[6] Ryan LMWD (2003). Post-concussion syndrome. Int Rev Psychiatry.

[7] Manuals M (2003). Concussion. Online medical library.

[8] Iverson GLLR (2003). Examination of postconcussion-like symptoms in a healthy sample. Appl Neuropsychol.

[9] Sivák S, Kurca E, Jancovic D, KP PS (2005). Contemporary view on mild brain injuries in adult population. Casopis lekaru ceskych.

[10] Hall RC, Hall RCCM (2005). Definition, diagnosis, and forensic implications of post-concessionalsyndrome. Psychosomatics.

[11] Barth JT, EP- P RR (2006) Mild traumatic brain injury: Definitions, Psychological Knowledge in Court: PTSD. Pain and TBI 46(3):271–277

[12] RA B (2008). Disentangling mild traumatic brain injury and stress reactions. N Engl J Med.

[13] Lee Lois K. (2007). Controversies in the Sequelae of Pediatric Mild Traumatic Brain Injury. Pediatric Emergency Care.

[14] Al J (2014). Et. The epidemiology of hospital-referred head injury. J Sci Res Reports.

[15] Bergersen K, Halvorsen JØ, Tryti EA, Taylor SI, Olsen A (2017). A systematic literature review of psychotherapeutic treatment of prolonged symptoms after mild traumatic brain injury. Brain Inj.

[16] Kifle Woldemichael1 N (2011). Magnitude and pattern of injury. Ethiop J Heal Sci.

[17] Crawford S, Wenden FJWD (1996). The Rivermead head injury follow up questionnaire: a study of a new rating scale and other measures to evaluate outcome after head injury. J Neurol Neurosurg Psychiatry.

[18] King NS, Crawford S, Wenden FJ, Moss NE, Wade DT (1995). The Rivermead post concussion symptoms questionnaire: A measure of symptoms commonly experienced after head injury and its reliability. J Neurol.

[19] Barker-Collo S, Theadom A, Jones K, Ameratunga S, Starkey N, Dudley M (2016). Reliable individual change in post concussive symptoms in the year following mild traumatic brain injury: data from the longitudinal, population-based brain injury incidence and outcomes New Zealand in the community (bionic) study. JSM Burn Trauma.

[20] Awang, Mohamed Saufi BB, Hup CK, Rus RM Post concussion syndrome after mild traumatic brain injury in a single neurosurgical center in East Coast, Malaysia-a preliminary report. Clin Med (Northfield Il) 2016, 15:2016

[21] Lannsjö M, Af Geijerstam JL, Johansson U, Bring J, Borg J (2009). Prevalence and structure of symptoms at 3 months after mild traumatic brain injury in a national cohort. Brain Inj.

[22] Saulle M, Greenwald BD (2012) Chronic traumatic encephalopathy: a review. Rehabil Res Pract. 2012:816069 10.1155/2012/816069 PMC3337491 22567320

