# Supplementary material for: Post-concussion syndrome among patients experiencing head injury attending emergency department of Hawassa University Comprehensive specialized hospital, Hawassa, southern Ethiopia
Source: J Headache Pain. 2018 Nov 21;19(1):112. doi: 10.1186/s10194-018-0945-0 (PMC6755541; doi:10.1186/s10194-018-0945-0)
Supplement: Supplementary file 1 — Social support of study participants attending emergency outpatient department of Hawassa University comprehensive specialized hospital Hawassa, Southern Ethiopia (n = 289) (DOCX 25 kb) [file 10194_2018_945_MOESM1_ESM.docx]

Additional File 1. Social support of study participants attending emergency outpatient department of Hawassa University compressive specialized hospital Hawassa, Southern Ethiopia (n=289)
