# Supplementary material for: Post-concussion syndrome among patients experiencing head injury attending emergency department of Hawassa University Comprehensive specialized hospital, Hawassa, southern Ethiopia
Source: J Headache Pain. 2018 Nov 21;19(1):112. doi: 10.1186/s10194-018-0945-0 (PMC6755541; doi:10.1186/s10194-018-0945-0)
Supplement: Supplementary file 2 — Cause of injures are the study participant attending emergency outpatient department of Hawassa university comprehensive specialized hospital Hawassa, Southern Ethiopia (n = 289) (DOCX 24 kb) [file 10194_2018_945_MOESM2_ESM.docx]

Additional File 2: Cause of injures among the study participant attending emergency outpatient department of Hawassa university comprehensive specialized hospital Hawassa, Southern Ethiopia (n=289)
