# Supplementary material for: Post-concussion syndrome among patients experiencing head injury attending emergency department of Hawassa University Comprehensive specialized hospital, Hawassa, southern Ethiopia
Source: J Headache Pain. 2018 Nov 21;19(1):112. doi: 10.1186/s10194-018-0945-0 (PMC6755541; doi:10.1186/s10194-018-0945-0)
Supplement: Supplementary file 3 — Symptoms of PCS of study participants attending emergency outpatient department of Hawassa University comprehensive specialized hospital, Hawassa, Southern Ethiopia (n = 289) (DOCX 16 kb) [file 10194_2018_945_MOESM3_ESM.docx]

Additional File 3: Symptoms of PCS among study participants attending emergency outpatient department of Hawassa University comprehensive specialized hospital, Hawassa, Southern Ethiopia (n=289)

| **Symptoms** | **Yes (%)** | **No (%)** |
| --- | --- | --- |
| Headaches | 227 (82.5) | 48 (17.5) |
| Feelings of dizziness | 2 (0.7) | 273 (99.3) |
| Nausea and/or vomiting | 131 (47.6) | 144 (52.4) |
| Noise sensitivity | 67 (24.4) | 208 (75.6) |
| Sleep disturbance | 116 (42.2) | 159 (57.8) |
| Fatigue, tiring more easily | 0 (0.0) | 275 (100) |
| Being irritable, easily angered | 8 (2.9) | 267 (97.1) |
| Feeling depressed or tearful | 4 (1.5) | 271 (98.5) |
| Feeling frustrated or impatient | 2 (0.7) | 273 (99.3) |
| Forgetfulness, poor memory | 1 (0.4) | 274 (99.6) |
| Poor concentration | 2 (0.7) | 273 (99.3) |
| Taking longer to think | 2 (0.7) | 273 (99.3) |
| Blurred vision | 3 (1.1) | 272 (98.9) |
| Light sensitivity | 5 (1.8) | 270 (98.2) |
| Double vision | 0 (0.0) | 275 (100) |
| Restlessness | 46 (16.7) | 229 (83.3) |
